# *In Vitro* Assessment of Marine *Bacillus* for Use as Livestock Probiotics

**DOI:** 10.3390/md12052422

**Published:** 2014-04-30

**Authors:** Maria Luz Prieto, Laurie O’Sullivan, Shiau Pin Tan, Peter McLoughlin, Helen Hughes, Montserrat Gutierrez, Jonathan A. Lane, Rita M. Hickey, Peadar G. Lawlor, Gillian E. Gardiner

**Affiliations:** 1 Eco-Innovation Research Centre, Department of Chemical and Life Sciences, Waterford Institute of Technology, Waterford, Ireland; E-Mails: 20038355@mail.wit.ie (M.L.P.); laurie.os@gmail.com (L.O.S.); 20039108@mail.wit.ie (S.P.T.); pmcloughlin@wit.ie (P.M.); hhughes@wit.ie (H.H.); 2 Veterinary Public Health Regulatory Laboratory, Department of Agriculture, Food and the Marine, Backweston Complex, Celbridge, Co. Kildare, Ireland; E-Mail: MM.Gutierrez@agriculture.gov.ie; 3 Teagasc Food Research Centre, Moorepark, Fermoy, Co. Cork, Ireland; E-Mails: jonathan.lane@teagasc.ie (J.A.L.); Rita.Hickey@teagasc.ie (R.M.H.); 4 Pig Development Department, Animal and Grassland Research and Innovation Centre, Teagasc, Moorepark, Fermoy, Co. Cork, Ireland; E-Mail: peadar.lawlor@teagasc.ie

**Keywords:** spores, antimicrobial, *E. coli*, xCELLigence, pigs

## Abstract

Six antimicrobial-producing seaweed-derived *Bacillus* strains were evaluated *in vitro* as animal probiotics, in comparison to two *Bacillus* from an EU-authorized animal probiotic product. Antimicrobial activity was demonstrated on solid media against porcine *Salmonella* and *E. coli.* The marine isolates were most active against the latter, had better activity than the commercial probiotics and *Bacillus pumilus* WIT 588 also reduced *E. coli* counts in broth. All of the marine *Bacillus* tolerated physiological concentrations of bile, with some as tolerant as one of the probiotics. Spore counts for all isolates remained almost constant during incubation in simulated gastric and ileum juices. All of the marine *Bacillus* grew anaerobically and the spores of all except one isolate germinated under anaerobic conditions. All were sensitive to a panel of antibiotics and none harbored *Bacillus* enterotoxin genes but all, except *B. pumilus* WIT 588, showed some degree of β-hemolysis. However, trypan blue dye exclusion and xCELLigence assays demonstrated a lack of toxicity in comparison to two pathogens; in fact, the commercial probiotics appeared more cytotoxic than the majority of the marine *Bacillus*. Overall, some of the marine-derived *Bacillus*, in particular *B. pumilus* WIT 588, demonstrate potential for use as livestock probiotics.

## 1. Introduction

In the past, sub-therapeutic doses of antibiotics were used in feed to promote growth and maintain health in farm animals, mainly pigs and poultry. However, due to concerns over increasing bacterial antibiotic resistance, the routine use of in-feed antibiotics was banned in the European Union (EU) in 2006. As a result, alternatives are now being sought. Probiotics, defined as “live microorganisms, which when administered in adequate amounts, confer a health benefit on the host” [1] are one such alternative. In fact, different probiotic strains have been shown to improve growth performance and to reduce enteric disease in pigs, poultry, ruminants and cultured fish [2,3].

In the EU, animal probiotics are controlled by Regulation EC 1831/2003, which establishes procedures for the authorization of feed additives [4]. This regulation and associated European Food Safety Authority (EFSA) guidelines [5] make no recommendations on strain origin, apart from the fact that the origin must be stated. However, it is generally recommended that probiotics should be isolated from the gastrointestinal tract (GIT) [6], as it is considered to give them the best chance of surviving in and colonizing the intestine [7]. Nonetheless, regardless of origin, *Bacillus* spores can tolerate the harsh environment of the GIT and are capable of germinating and proliferating within the intestine [8,9]. Furthermore, EU-authorized animal probiotics with proven efficacy are often not of intestinal origin. For example, six of the seven *Bacillus*-containing probiotic products currently licensed in the EU for use as feed additives, contain isolates from soil or soybean fermentations; in fact only one (*Bacillus subtilis* ATCC PTA-6737) is of intestinal origin [10,11,12,13,14,15,16].

Alternative probiotic sources should therefore not be overlooked. The marine environment is worth considering in this respect, given that it represents an untapped source of potentially novel microorganisms and that antimicrobial production, considered an important probiotic trait [17], is common amongst marine microflora [18]. Furthermore, *Bacillus*, which offers advantages as an animal probiotic, in that its spores survive harsh conditions, such as those encountered during feed manufacture and in the GIT, is commonly isolated from marine sources [3,19]. However, while marine-derived bacteria (mainly isolated from shrimp or fish intestines) have demonstrated beneficial effects when used as probiotics for fish [3], to our knowledge, they have not been evaluated as livestock probiotics to date.

As with any probiotic candidate organisms, rigorous *in vitro* testing is required prior to progressing to *in vivo* trials. The objective of the present study was, therefore, to characterize six seaweed-derived *Bacillus* isolates *in vitro*, for potential use as animal probiotics, using two *Bacillus* isolates from an EU-authorized animal probiotic product for comparison. The isolates were selected as most promising from a previous screening study on the basis of their antimicrobial activity against *Escherichia* *coli* and *Salmonella* [20], and in the present study were investigated mainly for their potential as probiotics for pigs.

## 2. Results

### 2.1. Molecular Fingerprinting and Growth of Marine Bacillus

The spontaneous rifampicin-resistant (Rif^r^) variants of the marine *Bacillus* yielded pulsed field gel electrophoresis (PFGE) fingerprints that were indistinguishable from those of the corresponding parent strains and their growth curves were also identical (data not shown). They were therefore used in all subsequent experiments. PFGE fingerprints of the marine *Bacillus* also differed from those of the two *Bacillus* isolates from the Bioplus 2B^®^ animal probiotic product, indicating that they were different strains (data not shown).

### 2.2. Sporulation Efficiency

As *Bacillus* probiotics will most likely be used as spore preparations, it is important to obtain information on sporulation. Sporulation is assumed to start at the end of the exponential phase in exhaustion medium. This occurred 12 h post-inoculation for *Bacillus pumilus* WIT 582, 588 and 592 and the two Bioplus 2B^®^ isolates, and after 15 h for *B. pumilus* WIT 584 and 590 and *Bacillus licheniformis* WIT 586 (data not shown). All of the marine isolates showed good sporulation efficiencies, with spore titres in the order of 10^7^–10^8^ spores/mL (data not shown). *B. licheniformis* WIT 586 and *B. pumilus* WIT 582 and WIT 590 had the highest sporulation rates (80%–82%), while *B. pumilus* WIT 588 had the lowest at 61%. In comparison, one of the commercial probiotics (*B. licheniformis* DSM 5749) had the highest sporulation efficiency (97%), while the other (*B. subtilis* DSM 5750) had one of the lowest (67%).

### 2.3. Growth and Spore Germination under Anaerobic Conditions

**Figure 1:**
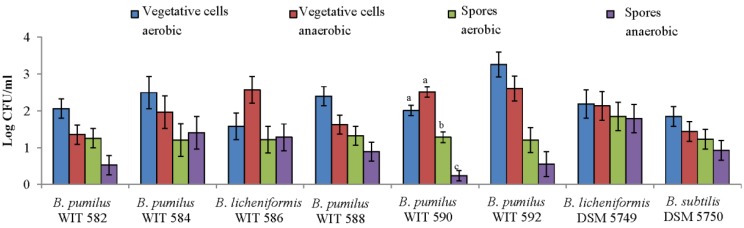
Increase in *Bacillus* counts following incubation at 37 °C for 24 h under aerobic or anaerobic conditions when inoculated into Brain Heart Infusion (BHI) broth as vegetative cells or spores at an inoculum of 10^6^–10^7^ colony forming units (CFU) or spores/mL. Values are the mean of data from triplicate experiments with SE indicated by vertical bars. Bars that do not share a common letter are signiﬁcantly different (*p* < 0.001). Where no letters are assigned to bars, there were no significant differences.

Anaerobic growth of the *Bacillus* isolates was investigated when either vegetative cells or spores were used as the inoculum (Figure 1), as anaerobic conditions prevail in the distal GIT. Using a spore inoculum, the increase in counts of *B. pumilus* WIT 590 was higher under aerobic than anaerobic conditions (*p* < 0.001). In fact, the increase in counts was very low under anaerobic conditions, indicating poor (if any) spore germination. Furthermore, the increase in counts was higher when vegetative cells were used as the inoculum, either under aerobic or anaerobic conditions, compared to spores (*p* < 0.001). However, all of the other *Bacillus* grew as well aerobically as they did anaerobically, both when spores and vegetative cells were used as the inoculum, and their spores all appear capable of germinating under anaerobic conditions.

### 2.4. Antimicrobial Activity on Solid Media

Although the marine *Bacillus* have been shown to have antimicrobial activity against a range of Gram-positive and some Gram-negative bacteria due, at least in part, to the production of bacteriocins [20], more targeted testing against pathogens of relevance to pigs was performed in the present study. Activity was determined against *Salmonella* Typhimurium and Derby (Table 1), as *Salmonella* transmission to humans via carcass contamination at slaughter is a major food safety issue and these serotypes are amongst those most commonly carried by pigs [21]. They are also capable of causing clinical disease. Similarly, the enterotoxigenic and enterohemorrhagic *E. coli* used as targets (Table 1) are pathogenic to animals, mainly pigs, and/or humans.

**Figure 2:**
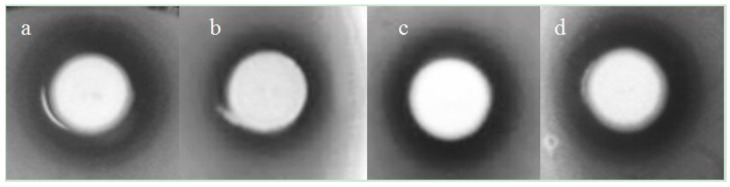
Representative examples of antimicrobial activity among *Bacillus* isolates. Activity was measured by deferred antagonism assays, where the marine isolates (**a**) *B. pumilus* WIT 582; (**b**) *B. pumilus* WIT 584; (**c**) *B. licheniformis* WIT 586 and (**d**) *B. pumilus* WIT 588) were grown on BHI agar prior to being overlaid with *E. coli* F3P3. Novobiocin was added to the overlay to prevent overgrowth of *Bacillus.*

*B. pumilus* WIT 582 had the best antimicrobial activity, with activity against all of the *E. coli* and *Salmonella* strains against which it was tested (Table 1, Figure 2). *B. licheniformis* WIT 586 and *B. pumilus* WIT 588 were active against all of the *E. coli* strains, and the latter also showed consistent activity against one *S. Typhimurium* isolate (Table 1, Figure 2). The other three marine bacteria had only weak antimicrobial activity, showing consistent activity against only one *E. coli* strain (WIT 584) or only variable activity against *S.* *Typhimurium* (WIT 590 and 592) (Table 1, Figure 2). In comparison, of the two *Bacillus* strains from the commercially available Bioplus 2B^®^ animal probiotic product, only *B. licheniformis* DSM 5749 showed activity and it was only consistently active against one *S. Typhimurium* strain (Table 1). To investigate potential activity against the marine pathogen *Vibrio*, the potential probiotics were also tested against *Vibrio cholerae* and *fischeri*. *B. pumilus* WIT 582, WIT 588 and *B. licheniformis* WIT 586 were active against the former, but none inhibited the latter (data not shown). In general, antimicrobial activity against Gram-negative bacteria (both those tested in the present and previous studies [20]) was detected only from growing cultures and not in the cell-free supernatant (data not shown)*.* All of the marine isolates also showed activity against four or five *Lactobacillus* species likely to be found in the porcine intestine but this activity was detected in the cell-free supernatants (Supplementary Information, Table S1. In comparison, the Bioplus 2B^®^ isolates showed greater activity against *Lactobacillus*, inhibiting nine and 14 species, respectively out of a total of 19 (Supplementary Information, Table S1).

**Table 1:** Antimicrobial activity ^1^ against porcine *E. coli* and *Salmonella* of marine *Bacillus* compared with that of *Bacillus* isolated from a commercially available animal probiotic product.

Test strain	*E. coli*						*Salmonella*				
	K88	F18ab	F2S2	F3P3	F1L3	F15OF3	Typhimurium DT104 (DPC 6046)	Typhimurium PT12 (DPC 6465)	Typhimurium DT17 (WIT 396)	Typhimurium DT104 (WIT 387)	Derby (WIT 411)
*B. pumilus* WIT 582	+	+++	++	++	++	+	++	+	+	++	++
*B. pumilus* WIT 584	-	-	-	++	-	+/−	-	-	-	-	-
*B. licheniformis* WIT 586	+	+	+	++	++	++	+/−	+/−	-	-	+/−
*B. pumilus* WIT 588	+++	+++	++	+++	+++	++	++	+/−	-	-	-
*B. pumilus* WIT 590	-	-	-	-	-	-	+/−	-	-	-	-
*B. pumilus* WIT 592	-	-	-	-	-	-	-	+/−	-	-	-
*B. licheniformis* DSM 5749 ^2^	-	-	-	-	-	-	+/−	++	-	-	-
*B. subtilis* DSM 5750 ^2^	-	-	-	-	-	-	-	-	-	-	-

^1^ Mean radii of zones of inhibition from triplicate deferred antagonism assays. + = 0.1–1 mm, ++ = 1.1–2 mm, +++ = 2.1–3 mm, ++++ >3 mm; - = no antimicrobial activity; +/− = variable activity; ^2^ Isolated from Bioplus 2B^®^.

### 2.5. Antimicrobial Activity in Liquid Culture

**Figure 3:**
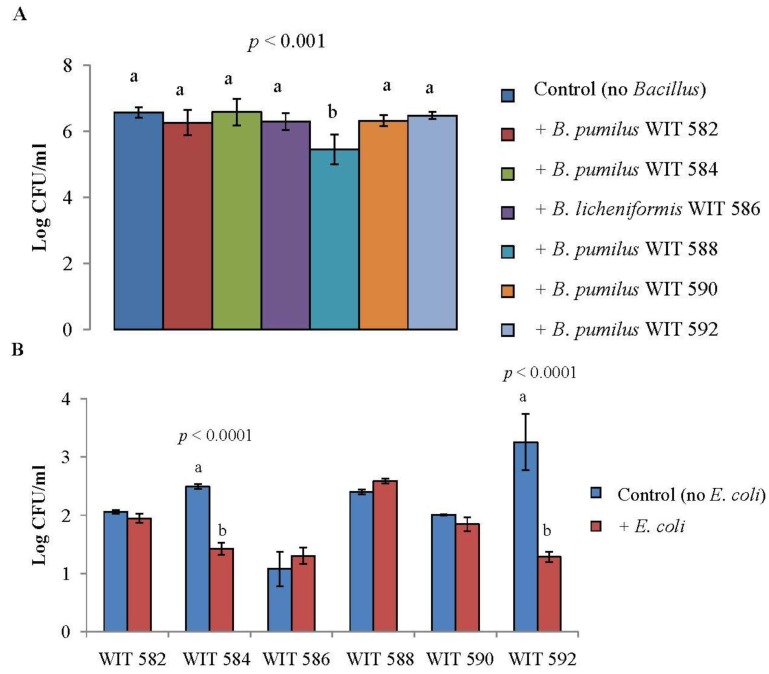
(**A**) Increase in *E. coli* DSM 10720 counts following co-incubation with vegetative cells of *Bacillus pumilus* WIT 582, 584, 588, 590 and 592 or *Bacillus licheniformis* WIT 586 at 37 °C for 24 h under aerobic conditions in BHI broth. The *E. coli* inoculum used was ~10^2^–10^3^ CFU/mL and for the *Bacillus* it was ~10^6^–10^7^ CFU/ml; (**B**) Increase in *Bacillus* counts following co-incubation with *E. coli* DSM 10720 at 37 °C for 24 h under aerobic conditions in BHI broth. Values are the mean of data from triplicate experiments with SE indicated by vertical bars. Bars that do not share a common letter are signiﬁcantly different. Where no letters are assigned to bars, there were no significant differences.

Firstly, the effects of co-incubation of spores of the marine *Bacillus* with either *S. Typhimurium* or *E. coli* were studied in liquid culture anaerobically (to simulate intestinal conditions). No reduction in *Salmonella* or *E. coli* counts was observed compared to controls without *Bacillus* spores (*p* > 0.05; data not shown), even though the *Bacillus* grew as well with or without *E. coli*/*Salmonella* (*p* > 0.05; data not shown). The co-culture experiments were repeated with a lower inoculum of *Salmonella/E. coli* (*i.e.*, ~10^2^–10^3^ instead of ~10^6^–10^7^ CFU/mL). However, no reductions in either *Salmonella* or *E. coli* counts were obtained, even though the *Bacillus* isolates again grew as well with or without the Gram-negative bacteria (*p* > 0.05; data not shown). The co-incubation of *Bacillus* vegetative cells with *Salmonella* (data not shown) and *E. coli* (Figure 3) was also studied under aerobic conditions to overcome possible issues with spore germination and to provide the *Bacillus* isolates with more favorable growing conditions. Again, no reductions in *Salmonella* counts were obtained, even though the *Bacillus* grew as well when co-cultured with *Salmonella* as when grown alone (*p* > 0.05; data not shown). However, when the same experiment was conducted with *E. coli*, the increase in *E. coli* counts was ~10-fold less when co-cultured with *B.* *pumilus* WIT 588 (*p* < 0.001; Figure 3A). The *Bacillus* grew as well with and without *E. coli*, except for *B. pumilus* WIT 584 and WIT 592 which had less of an increase in counts when co-cultured (*p* < 0.0001; Figure 3B).

### 2.6. Bile Tolerance and Resistance to Simulated Gastrointestinal Conditions

Ability to survive the harsh environment in the stomach and small intestine is one of the properties required of probiotics. Bile tolerance was first investigated, as bile is a bactericidal agent found in the small intestine. Three of the marine isolates (*B. pumilus* WIT 582 and 588 and *B. licheniformis* 586) and one of those isolated from the commercial probiotic product (*B. subtilis* DSM 5750) grew at concentrations of up to 1% bile, while *B. licheniformis* DSM 5749 was capable of growth up to 2% (Table 2). Given the origin of the isolates, salt tolerance was also investigated and compared with that of the commercial probiotics. Even though it is not particularly relevant for animal probiotics, salt tolerance may be important for probiotic survival in certain foods. All of the isolates, except *B. pumilus* WIT 584 and *B. licheniformis* WIT 586, were capable of growing at concentrations of up to 10% NaCl (data not shown) and can therefore, be considered salt tolerant. Interestingly, this included the commercial probiotics which are not of marine origin. However, none of the isolates was capable of growth at the maximum concentration investigated (15%).

**Figure 4:**
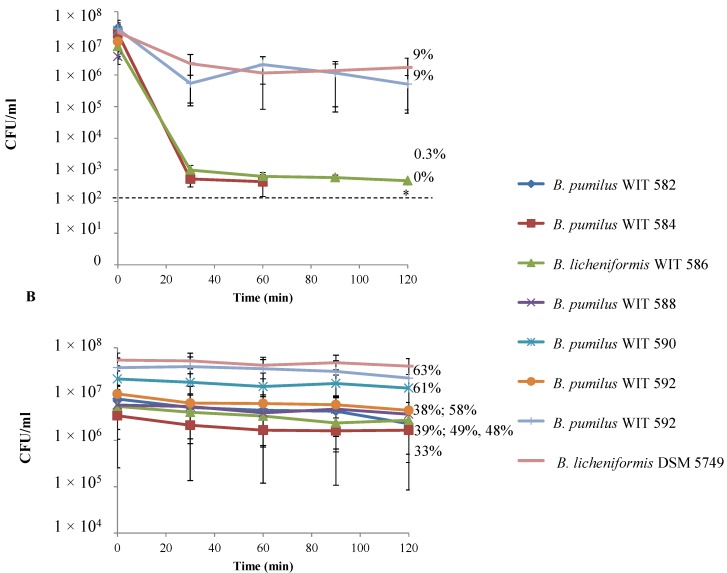
Survival of *Bacillus* isolates in simulated gastric juice, pH 2.2, over time when inoculated as (**A**) vegetative cells or (**B**) spores. Values are the mean of data from triplicate experiments with SE indicated by vertical bars. Percent survival after 2 h is also indicated. * = the limit of detection, which was 100 CFU/mL (values below the limit of detection were recorded as 100 CFU/mL).

**Figure 5:**
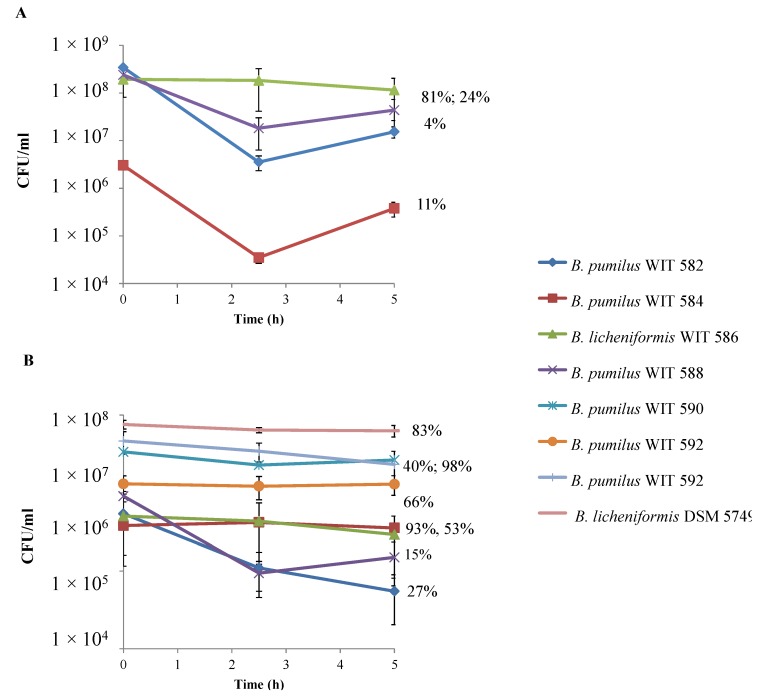
Survival of *Bacillus* isolates in simulated ileum juice, pH 7, over time when inoculated as (**A**) vegetative cells or (**B**) spores. Only vegetative cells of WIT 584, WIT 586, DSM 5749 and DSM 5750 were tested, as they were the only isolates to survive simulated gastric juice. Values are the means of data from triplicate experiments with SE indicated by vertical bars. Percent survival after 5 h is also indicated.

*Bacillus* spores and vegetative cells were also tested for resistance to simulated gastric juice (pH 2.2) and simulated ileum juice (pH 7) (Figure 4 and Figure 5). After only 30 min of exposure to simulated gastric juice, vegetative cells of *B. pumilus* WIT 582, WIT 588, WIT 590 and WIT 592 were undetectable (Figure 4A). On the other hand, *B. pumilus* WIT 584, although reduced by ~4 log CFU/mL, was detectable after 1 h at a survival rate of 0.2%, but not at 90 min. *B. licheniformis* WIT 586 was the most tolerant of the marine isolates and, again although the count was reduced dramatically, it was capable of surviving for up to 2 h under simulated gastric conditions, albeit at a low rate (0.3%). However, the *Bacillus* isolated from Bioplus 2B^®^ (*B. licheniformis* DSM 5749 and *B. subtilis* DSM 5750) were the most resistant; after an initial 1.4–1.6 log CFU/mL decline, counts remained relatively stable over the remainder of the 2-h incubation period, with a survival rate of 9% obtained for both strains. Only those isolates that demonstrated some degree of resistance to simulated gastric juice (*B. pumilus* WIT 584, *B. licheniformis* WIT 586 and DSM 5749 and *B. subtilis* DSM 5750) were selected for study in simulated ileum juice. Counts of all isolates declined by 1–2 log CFU/mL after 2.5 h but increased or remained stable thereafter (Figure 5A). As a result, final counts recovered after the 5-h incubation period were only slightly lower than initial counts, with survival rates ranging from 4% to 81%. In comparison to vegetative cells, spores of all isolates were more resistant to both simulated gastric and ileum juices. This was evidenced by the fact that counts remained relatively stable during the 2- and 5-h treatment periods, respectively, with maximum survival rates of 63% and 98% obtained (Figure 4B and Figure 5B).

**Table 2:** *In vitro* assessment of probiotic properties of marine *Bacillus* compared with *Bacillus* isolated from a commercially available animal probiotic product and a *Lactobacillus* probiotic.

Test strain	Swarming motility ^1^	Bile tolerance ^2^	Adherence to intestinal epithelial cells ^3^
*B. pumilus* WIT 582	+	1%	17.4 ± 8.3 ^a^
*B. pumilus* WIT 584	−	<0.3%	12.1 ± 10.6 ^a^
*B. licheniformis* WIT 586	+	1%	11.9 ± 5.1 ^a^
*B. pumilus* WIT 588	−	1% ^4^	9.3 ± 4.0 ^a^
*B. pumilus* WIT 590	−	<0.3%	11.0 ± 10.5 ^a^
*B. pumilus* WIT 592	+	<0.3%	268.4 ± 239.7 ^b^
*B. licheniformis* DSM 5749	+	2% ^4^	116.5 ± 79.9 ^b^
*B. subtilis* DSM 5750	+	1% ^4^	196.6 ± 87.1 ^b^
*Lb. rhamnosus* GG	ND ^5^	ND	705.6 ± 182.8 ^b^

^1^ + = swarming motility (indicated by growth of the isolate over the entire surface of the plate); − = absence of swarming motility (from triplicate assays); ^2^ maximum concentration of bile at which growth was observed. Growth was assessed in triplicate on BHI agar containing 0.3, 0.5, 1 and 2% (*w*/*v*) porcine bile after 48 h; ^3^ bacterial cells/100 HT-29 intestinal epithelial cells (mean of triplicate assays ± SE) after 4 h of co-incubation; ^4^ *B.* pumilus WIT 588 and *B. subtilis* DSM 5750 grew at a concentration of up to 0.5% bile after 24 h and 1% after 48 h. *B. licheniformis* DSM 5749 grew at a concentration of up to 0.5% after 24 h and 2% after 48 h; ^5^ ND = not determined; ^a, b^ values within a column without a common letter are significantly different (*p <* 0.05).

### 2.7. Biofilm Formation, Swarming Motility and Adherence to Intestinal Epithelial Cells

Biofilm formation was assessed in all *Bacillus* isolates, as it may facilitate adherence to intestinal epithelial cells [22] and could therefore, be considered a probiotic property. All isolates, both marine-derived and those isolated from Bioplus 2B^®^, were capable of forming biofilms *in vitro* (data not shown). Swarming motility was also investigated as it may also assist in intestinal colonization [23]. Swarming motility was observed in three of the marine isolates and both of those isolated from the Bioplus 2B^®^ product (Table 2).

Adherence of the marine and Bioplus 2B^®^ isolates to intestinal epithelial cells was also investigated and compared to an adherent *Lactobacillus* probiotic (*Lactobacillus rhamnosus* GG) (Table 2). When presented as the number of bacterial cells adhering to 100 epithelial cells, adherence rates did not differ between *B. pumilus* WIT 592, the Bioplus 2B^®^ isolates (*B. subtilis* DSM 5750, *B. licheniformis* DSM 5749) and the positive control *Lb. rhamnosus* GG (*p* *>* 0.05). Furthermore, these isolates demonstrated higher rates of adherence than the other five marine-derived *Bacillus* strains (*p* < 0.05). When expressed as percent adherence (*i.e.*, the number of bacterial cells adhering to the epithelial cells relative to the total number added), rates of 0.001%–0.06% were obtained for the *Bacillus* isolates and no strain differences were observed (*p* > 0.05). However, *Lb. rhamnosus* GG displayed a higher adherence rate (0.305%; *p* < 0.05; data not shown).

### 2.8. In Vitro Safety Testing of Bacillus Isolates

#### 2.8.1. Hemolytic Activity, Antibiotic Resistance and Presence of Enterotoxin Genes

Data on hemolytic activity of the *Bacillus* isolates, as determined on sheep blood agar, are presented in Table 3. Only two of the marine isolates (*B. pumilus* WIT 582 and *B. licheniformis* WIT 586) showed complete β-hemolysis and three others (*B. pumilus* WIT 584, 590 and 592) were only weakly or very weakly β-hemolytic. *B. pumilus* WIT 588 and the two Bioplus 2B^®^ isolates did not display any hemolytic activity. All of the *Bacillus* isolates were sensitive to the panel of nine antibiotics against which they were tested, as the minimum inhibitory concentration (MIC) values were lower than the breakpoints set by EFSA [24] (data not shown). We also tested for the presence of genes encoding *Bacillus cereus* enterotoxins, as although none of the isolates are *B. cereus*, Fakhry *et al.* [25] showed that other species can harbor these genes. All of the isolates were negative in the PCR analysis (data not shown), demonstrating that they did not harbor genes for any of these known *Bacillus* enterotoxins.

**Table 3:** Safety testing of marine *Bacillus* compared with *Bacillus* isolated from a commercially available animal probiotic product and a *Lactobacillus* probiotic.

Test strain	Viability of intestinal epithelial cells (%) ^1^	Hemolysis ^2^
*B. pumilus* WIT 582	65.9 ± 3	β
*B. pumilus* WIT 584	53 ± 10	very weak β
*B. licheniformis* WIT 586	80.7 ± 4	β
*B. pumilus* WIT 588	69.8 ± 3	γ
*B. pumilus* WIT 590	86.2 ± 7	very weak β
*B. pumilus* WIT 592	91.2 ± 6	weak β
*B. licheniformis* DSM 5749	74.9 ± 6	γ
*B. subtilis* DSM 5750	92.4 ± 10	γ
*Lb. rhamnosus* GG	68.6 ± 5	ND

^1^ Viability of HT-29 intestinal epithelial cells (mean of triplicate assays ± SE) compared to the control without bacteria (set at 100% via7bility) as measured by trypan blue staining following 5 h of co-incubation; ^2^ Incubated on 5% sheep blood agar at 30 °C for 3 days. β = complete hemolysis, with clear zones around each colony; γ = no hemolysis (from triplicate assays).

#### 2.8.2. Cytotoxic Activity against Intestinal Epithelial Cells

All of the *Bacillus* isolates grew to concentrations of up to 10^7^–10^9^ CFU/mL in the cell culture (CC) medium after 24 h, demonstrating that they were capable of growing well in this medium. The effect of the marine-derived and probiotic *Bacillus* and the probiotic *Lactobacillus* on viability of the HT-29 intestinal epithelial cells, as measured by trypan blue staining are shown in Table 3. Two of the marine *Bacillus* (WIT 590 and WIT 592) and one of the *Bacillus* probiotics (DSM 5750) caused only minimal (7.6%–13.8%) reductions in cell viability. On the other hand, the other marine *Bacillus*, one of the probiotic *Bacillus* and the *Lactobacillus* probiotic caused reductions of 19.3%–47%.

The xCELLigence real-time cell analysis system was then used to obtain more detailed data on any potential toxic effects of the candidate probiotics on intestinal epithelial cells. The optimal concentration of HT-29 cells was first determined. As illustrated in Supplementary Figure S1, a density of 20,000 cells/mL had the greatest slope in combination with the shortest doubling time, indicating that the cells were in the exponential phase of growth. As the HT-29 cells must be growing exponentially prior to bacterial exposure, this seed number was therefore used in subsequent cytotoxicity assays.

Cytotoxicity of the marine *Bacillus* was assessed by taking impedance (cell index; CI) measurements over time. Proliferation of cells or cell spreading results in enhanced electrical impedance resulting in a higher cell index (CI) value [26]. The addition of the bacteria produced an initial increase in the CI, followed by a decrease at different rates, depending on the bacterial strain (Supplementary Information, Figure S2). The pathogenic methicillin sensitive *Staphylococcus aureus* (MSSA) and *E. coli* strains began to reduce the CI 6 h post-addition, while one of the animal probiotics (*B. licheniformis* DSM 5749) resulted in a decline after 9 h and the other (*B. subtilis* DSM 5750), together with the human probiotic *Lb. rhamnosus* GG, yielded reductions after 15 h (Supplementary Information, Figure S2). On the other hand, it was 22 h post-addition before any of the marine bacteria produced decreases in CI (Supplementary Information, Figure S2).

**Figure 6:**
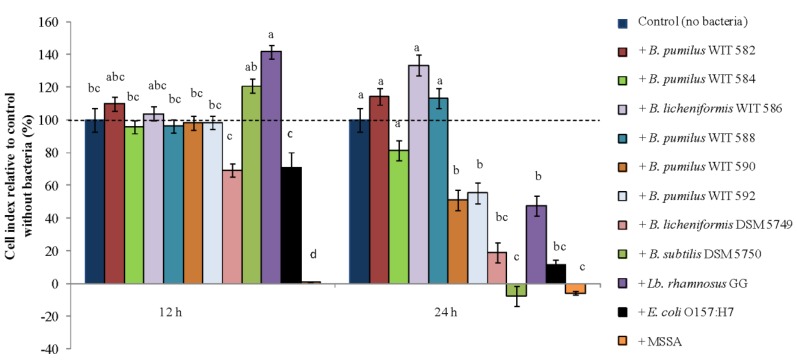
Cell index relative to the control without bacteria (which was set at 100%) of HT-29 intestinal epithelial cells measured using the xCELLigence real-time cell analysis system following 12 and 24 h of exposure to the marine *Bacillus* (WIT 582, 584, 586, 588, 590 and 592), *Bacillus* isolated from the Bioplus 2B^®^ probiotic product (DSM 5749 and 5750), a *Lactobacillus* probiotic (*Lb. rhamnosus* GG) and the pathogens *E. coli* O157: H7 NCTC 12900 and methicillin sensitive *Staphylococcus aureus* (MSSA). Values are the mean of data from triplicate experiments with SE indicated by vertical bars. Bars that do not share a common letter are significantly different (*p <* 0.001).

When the CI of the HT-29 cells co-incubated with the bacteria was calculated relative to that of control HT-29 cells without bacteria, the results showed that after 12 h of co-incubation the MSSA strain decreased the CI compared to the control and all other bacterial strains (Figure 6; *p* < 0.001) in agreement with the data reported above. The *E. coli* pathogen as well the *B. licheniformis* DSM 5749 probiotic also decreased the CI but only when compared with the probiotic *B. subtilis* and *Lb. rhamnosus* strains (Figure 6; *p* < 0.001). After 12 h the marine bacteria did not produce any differences in the CI of the HT-29 cells compared to the control without bacteria (Figure 6; *p* > 0.05). On the other hand, *Lb. rhamnosus* GG increased the CI in comparison to all except three treatments (*p* < 0.001; Figure 6).

After 24 h of co-incubation, the MSSA pathogen together with the *B. subtilis* probiotic decreased the CI relative to the control and all except two of the bacterial treatments (*p* < 0.001; Figure 6). In addition, the pathogenic *E. coli* strain as well as the marine bacteria, *B. pumilus* WIT 590 and WIT 592, and the probiotic *B. licheniformis* and *Lb. rhamnosus* strains decreased the CI relative to the control (*p* < 0.001; Figure 6). The remaining marine bacteria did not cause any differences compared to the control (*p* > 0.05; Figure 6).

## 3. Discussion

*Bacillus*, well-known for antimicrobial production, have other advantages as probiotics, as their spores survive harsh conditions, such as those encountered in the GIT and during product manufacture [19]. Although it is recommended, but not a prerequisite, that probiotics are sourced from the GIT, *Bacillus* are not considered endogenous intestinal microbiota. Indeed, *Bacillus* from sources as diverse as soil, fermented soybeans and Chinese herbs have proven efficacious as animal probiotics [10,11,12,14,16,27,28]. The marine environment is an untapped source of novel microorganisms in which antimicrobial production is a common trait [18] and from which *Bacillus* is commonly recovered [20,29]. However, although marine-derived *Bacillus* have been explored as probiotics for aquaculture [3,19], this is the first study to investigate their potential as livestock probiotics.

While the efficacy of animal probiotics must ultimately be demonstrated *in vivo* [5], initial *in vitro* tests are important for strain selection. The six seaweed-derived *Bacillus* investigated in the present study were selected based on antimicrobial activity against Gram-negative pathogens [21]. This may be due to the production of bacteriocins [20], as although *Bacillus* bacteriocins are reportedly more active against Gram-positive bacteria, some have a broader spectrum that also includes Gram-negatives [30,31]. However, additional probiotic properties and safety must be evaluated prior to *in vivo* use. This was the purpose of the present study, which used two proven *Bacillus* animal probiotics from an EU-authorized product (Bioplus 2B^®^) for comparison. Antimicrobial activity against a range of serotypes of *Salmonella* and *E. coli* (both zoonotic and animal pathogens) was first demonstrated on solid media for the majority of the marine isolates. More activity was observed against *E. coli*, in agreement with preliminary findings [20]. The reason for the poor anti-*Salmonella* activity is not known but might be due to the fact that *Salmonella* may be resistant to the particular bacteriocins or antimicrobials produced. Most of the marine isolates were more active against Gram-negatives than the isolates from the animal probiotic product but showed less activity against *Lactobacillus* and *Bifidobacterium*. The latter is desirable given that these genera are considered beneficial in the intestinal tract.

The fact that the marine *Bacillus* showed only weak activity against *Salmonella* in agar plate assays likely explains the lack of anti-*Salmonella* activity in liquid culture. However, all of the marine isolates previously showed activity against *E. coli* DSM 10720 on solid media [20] but only one (*B. pumilus* WIT 588) reduced counts of this *E. coli* strain in liquid culture. This may be explained by the fact that the antimicrobial(s) seems not to be released from the bacterial cells. Similarly, Ruiz-Ponte *et al.* [32] observed that a marine *Roseobacter* isolate with activity against *Vibrio pectenicida* did not reduce counts in co-incubation studies.

Two of the marine isolates were resistant to simulated gastric transit in the vegetative state, although the level of resistance was low and neither was as tolerant as the commercial probiotics. However, if vegetative cells were to be used as probiotics, delivery in a feed matrix would likely provide protection, as previously demonstrated for food [33]. Moreover, the spores of all isolates survived well, which is not surprising considering their resilience. In any case, it appears that once the marine *Bacillus* have transited the stomach, they will survive, at least in the small intestine, regardless of whether vegetative cells or spores are used. This seems to be the case even if the vegetative cells do not tolerate physiological concentrations of bile (0.3%) [34], as was the case for *B. pumilus* WIT 584.

The fact that all of the isolates formed biofilms *in vitro* and that some exhibit swarming motility may mean that they will be protected from harsh conditions in the GIT. These properties could also prevent pathogen attachment and facilitate intestinal colonization [23,25,35]. The latter is backed up by the fact that all of the marine *Bacillus* isolates were capable of adhering to colonic epithelial cells *in vitro*, although all except one were less adherent than the commercially available probiotics. Furthermore, our isolates showed low percent adherence rates, in agreement with those observed for probiotic *B. cereus* strains [36], but in contrast to those reported for *B. polyfermenticus* potential probiotics [37].

All of the marine *Bacillus* were also capable of growing anaerobically, in agreement with the fact that *B. pumilus* and *licheniformis* are facultative anaerobes. Anaerobic growth from vegetative cells was as good as that of the Bioplus 2B^®^ isolates, which have been shown to grow in the porcine GIT [38]. However, *Bacillus* feed additives are usually used as spores, mainly due to their resilience to gastrointestinal and manufacturing conditions and their extended shelf-life [19]. All of the marine *Bacillus* sporulated efficiently (at least as well as the Bioplus 2B^®^ isolates which are used commercially as a spore preparation). It is generally thought that *Bacillus* spores must be capable of germinating in the intestine in order to obtain a probiotic effect [39]. However, although spore germination is most likely necessary for certain probiotic effects, e.g., antimicrobial production, it may not be a prerequisite, as benefits such as immunomodulation and competitive exclusion could potentially be achieved by spores. Nonetheless, spores of all except one of the marine isolates were capable of germinating under anaerobic conditions, albeit cell numbers did not increase as much as for vegetative cells. This may be explained by the fact that spores need more time to germinate, and then grow than vegetative cells. In any case, anaerobic outgrowth from spores of some of the marine isolates was as good as that of the Bioplus 2B^®^ isolates, which have proven capable of germinating in the porcine GIT [38]. Taken together, these *in vitro* data suggest that at least some of the marine isolates would be able to survive gastrointestinal transit and subsequently germinate and grow in the mammalian GIT. However, animal feeding trials are required in order to definitively evaluate intestinal survival, as well as spore germination and outgrowth *in vivo* and these have been reported in a follow-on study published by our group [40].

However, even if micro-organisms demonstrate probiotic potential, they cannot be used in practice unless they are safe. Particular care is necessary when selecting *Bacillus* strains for use as probiotics, as there are specific concerns regarding the use of this genus, namely toxin production and dissemination of antibiotic resistance genes [29]. This is highlighted by the fact that toxigenic potential, hemolytic activity and high levels of antibiotic resistance have been found in commercial human *Bacillus* probiotics [41,42]. Although, *B. licheniformis* and *B. pumilus* are considered by EFSA to qualify for “qualified presumption of safety” (QPS) status, a generic qualification for all QPS bacterial taxonomic units is that strains should not harbor any acquired antimicrobial resistance genes to clinically relevant antibiotics [43]. For *Bacillus*, absence of toxigenic activity should also be demonstrated [44]. When tested in accordance with EFSA guidelines for *Bacillus* spp. used in animal nutrition [44] none of the marine isolates were found to harbor enterotoxin genes. Neither were they resistant to any of the antibiotics of human or veterinary importance set out in EFSA guidelines for microbial feed additives [24]. Hemolytic activity was also tested, as it has been found in commercial *Bacillus* probiotics [41] and it is a test recommended by EFSA to detect toxigenic potential [44]. *B. pumilus* WIT 588 was the only marine isolate that showed a complete lack of hemolytic activity, but three other isolates displayed only weak or very weak activity. While *in vitro* hemolytic activity does not always lead to negative effects *in vivo*, as evidenced by studies in fish and pigs [36,45], EFSA guidelines state that *Bacillus* strains proven to be hemolytic are not recommended for use as feed additives [44]. Therefore, it would be preferable to select only the non-hemolytic or perhaps weakly/very weakly hemolytic isolates from the present study for probiotic use.

Cytotoxicity testing is also recommended by EFSA to detect toxigenic potential of *Bacillus* feed additives [44]. Preliminary assays using the trypan blue dye exclusion assay showed that at least half of the marine *Bacillus* strains demonstrated some toxic effects against colonic epithelial cells *in vitro*. However, the dynamic real-time xCELLigence cell analysis system, used here for the first time to evaluate potential probiotics, demonstrated a lack of toxicity of any of the marine bacteria when compared with two known pathogens. Interestingly, the commercial strains (in particular the *B. licheniformis*) which have a safe history of use as probiotics [12,38] appeared to display greater cytotoxicity than the majority of the marine *Bacillus*. The greatest toxic effects were observed 24 h post-addition; however, effects observed at this time point could be due to nutrient exhaustion and may be unrelated to toxicity of the bacteria, highlighting the limitations of *in vitro* assays.

## 4. Experimental Section

### 4.1. Bacterial Strains and Culture Conditions and Chemicals

All chemicals and cell culture media were purchased from Sigma Aldrich (Dublin, Ireland) unless otherwise stated. Six seaweed-derived *Bacillus* isolates, *B. pumilus* WIT 561, WIT 563, WIT 572,WIT 573 and WIT 574 and *B. licheniformis* WIT 565, were selected as potential probiotics from a previous screening study on the basis of their antimicrobial activity against *E. coli* and *Salmonella* [20]. It should be noted that the WIT 561, 563, 573 and WIT 574 strains referred to as *B. pumilus* in fact belong to the *B. pumilus* group, as they cannot be distinguished from other members of this group (*Bacillus altitudinis*, *Bacillus aerophilus*, *acillus. safensis*, *Bacillus stratosphericus*) by 16S rRNA gene sequencing [20]. The WIT 572 strain has been confirmed as *B. pumilus* on the basis of sequencing of the *gyrB* and *pyrE* housekeeping genes [40]. Rif^r^ variants of each of the *Bacillus* isolates were generated in order to facilitate subsequent enumeration *in vivo* according to the method of Gardiner *et al.* [46] and were used in all experiments. The Rif^r^ variants were designated *B. pumilus* WIT 582, WIT 584, WIT 588, WIT 590 and WIT 592 and *B. licheniformis* WIT 586, respectively. To confirm that the Rif^r^ variants were identical to the parent strains, growth curves were performed by inoculating overnight cultures of each of the isolates (1%) into 250 µL of Brain Heart Infusion (BHI) broth (Oxoid Ltd., Basingstoke, Hampshire, UK) in 96-well plates and incubating at 37 °C with agitation. The OD_600_ was measured every 10 min for 24 h using a 96-well plate reader (BioTek Instruments, Winooski, VT, USA). Assays were performed in triplicate. Molecular fingerprinting by PFGE was also performed, as described previously [20]. *B. licheniformis* and *B. subtilis* were isolated from the Bioplus 2B^®^ animal probiotic product (Chr. Hansen, Hørsholm, Denmark) and their identity confirmed by 16S rRNA gene sequencing, as outlined previously [20]. PFGE fingerprints of the Rif^r^ variants of the marine bacteria were also compared with those of the Bioplus 2B^®^ isolates. The marine *Bacillus* and the *Bacillus* isolated from Bioplus 2B^®^ were routinely grown in/on BHI broth/agar at 37 °C, with agitation used for broth cultures. Bacterial strains used as indicators for detection of antimicrobial activity and as controls for hemolysis, enterotoxin gene, adhesion and cytotoxicity assays, and their growth conditions, are listed in Supplementary Information, Table S2.

### 4.2. Determination of Time Taken to Sporulate and Sporulation Efficiency and Preparation of Spores

The *Bacillus* cultures were induced to sporulate by the nutrient exhaustion method outlined by Nicholson and Setlow [47] except that 250 µL of cell suspension was added to a 96-well plate, and the OD_600_ was measured every 30 min for 48 h using a 96-well plate reader. The start of sporulation is arbitrarily defined as the time at which the cultures cease to grow exponentially in the exhaustion medium and was noted for each isolate. Cultures were harvested by centrifugation (10,000× *g*, 10 min) 48 h after inoculation and spores were purified using lysozyme treatment and salt and detergent washes as described by Nicholson and Setlow [47]. Spores were suspended in sterile deionized water at 4 °C and a final purification step was added, which involved heating the spore suspensions to 80 °C for 10 min to ensure elimination of all vegetative cells. Total viable counts of spore suspensions were determined by diluting 10-fold in maximum recovery diluent (MRD; Merck, Darmstadt, Germany) and spread plating on BHI agar incubated at 37 °C for 24 h. Purified spores were centrifuged (10,000× *g*, 10 min) and re-suspended in sterile deionized water at 4 °C. Sporulation efficiency was determined by inducing sporulation, as outlined above and, 48 h after inoculation, performing viable counts before and after heating to 80 °C for 20 min. Sporulation efficiency was calculated as the percentage of survivors remaining after heating.

### 4.3. Evaluation of Bacillus Growth and Spore Germination under Anaerobic Conditions

The ability of the *Bacillus* isolates to grow anaerobically was evaluated by inoculating an overnight culture of each (1%) into 5 mL BHI broth and incubating at 37 °C for 24 h aerobically with shaking at 200 rpm and anaerobically in anaerobic jars with CO_2_-generating kits (Anaerocult A™; Merck). Counts were performed initially (0 h) and after 24 h by diluting 10-fold in MRD, spread plating appropriate dilutions on BHI agar and incubating at 37 °C for 24 h. The ability of the spores to germinate under anaerobic conditions was also evaluated using the same procedure except that spore suspensions were used as the inoculum. Increases in *Bacillus* counts were calculated as follows:

Increase in *Bacillus* = Number of bacteria at 24 h (CFU/mL) − Number bacteria at 0 h (CFU/mL) (1)

All experiments were conducted in triplicate.

### 4.4. Assessment of Antimicrobial Activity of Bacillus Isolates on Solid Media

Antimicrobial activity against the *Salmonella*, *E. coli* and *Vibrio* strains listed in Supplementary Information, Table S2 was determined in triplicate using the deferred antagonism assay as outlined previously [20], with the *Bacillus* isolates spotted onto BHI agar and overlaid with the appropriate soft agar seeded with a 0.25% (*v*/*v*) inoculum of each of the indicator bacteria. Novobiocin (0.001% *w*/*v*) was added to the overlay to prevent overgrowth of *Bacillus*. Antimicrobial activity against these Gram-negative bacteria as well as the *Lactobacillus*, *Weissella* and *Bifidobacterium* strains listed in Supplementary Information, Table S2 was also determined by the well diffusion assay (WDA), as previously described [20].

### 4.5. Assessment of Antimicrobial Activity of Bacillus Isolates in Liquid Culture

In the first experiment, 10 mL BHI broth was inoculated to a final concentration of ~10^6^–10^7^ CFU/mL of *Salmonella* PT12 (DPC 6465) or *E. coli* DSMZ 10720 from an overnight culture. Each of the marine *Bacillus* isolates was then added as a spore suspension at a final concentration of ~10^6^–10^7^ spores/mL and the bacteria were co-incubated anaerobically at 37 °C. In the second experiment, the conditions were the same, except that the initial concentration of *Salmonella/E. coli* was ~10^2^–10^3^ CFU/mL. The third experiment was conducted in the same way but with an inoculum of ~10^7^ CFU/mL vegetative cells of *Bacillus* and ~10^2^–10^3^ CFU/mL *Salmonella* or *E. coli* incubated aerobically at 37 °C with agitation (200 rpm). In each experiment control cultures were inoculated with *Bacillus* spores/vegetative cells or *Salmonella/E. coli* alone, depending on the experiment. Samples were taken at 0 and 24 h. *Salmonella*, *E. coli* and *Bacillus* were enumerated by performing 10-fold serial dilutions in MRD and spread-plating appropriate dilutions on xylose lysine deoxycholate agar (XLD; Oxoid), ChromoCult^®^ tryptone bile X-glucuronide (CTBX; Merck) and BHI agar containing 100 μg/mL rifampicin, respectively. All plates were incubated at 37 °C for 24 h. Increases in *Salmonella*, *E. coli* and *Bacillus* counts were calculated as outlined in Section 4.3. All assays were performed either in duplicate or triplicate.

### 4.6. Bile, Salt Tolerance and Gastric and Ileum Juice Assays

Tolerance of the *Bacillus* isolates to porcine bile was assessed as described by Casey *et al.* [48], except that BHI agar with bile concentrations of 0.3, 0.5, 1.0 and 2.0% (*w*/*v*) was used and plates were incubated aerobically for 48 h. Salt tolerance was tested on BHI plates containing 5, 10 and 15% (*w*/*v*) NaCl, incubated at 37 °C for 48 h. For both bile and salt tolerance assays, plates were examined for evidence of bacterial growth after 24 h and 48 h and both assays were conducted in triplicate.

The survival of *Bacillus* spores and vegetative cells in simulated gastric and ileum juices was determined as described by Dobson *et al.* [49] with some modifications as follows; cells from overnight cultures were harvested by centrifugation at 14,000*×* *g* for 2 min, washed in MRD and adjusted to OD_600_ = 1. Spore suspensions were also prepared in this way. Each cell/spore suspension was added to simulated gastric (pH 2.2) or simulated ileum juice (pH 7.0) and incubated for 2 and 5 h, respectively. Samples were taken at regular intervals, and counts obtained by spread-plating on BHI agar incubated at 37 °C for 24 h. While the spores of all isolates were evaluated under simulated gastric and ileum conditions, only the vegetative cells of isolates that survived simulated gastric juice were evaluated in simulated ileum juice. All assays were performed in triplicate.

### 4.7. Determination of Biofilm Formation and Swarming Motility

Biofilm formation was tested as described by Barbosa *et al.* [35] except that overnight cultures of each of the *Bacillus* isolates were inoculated (1%) into 3 mL low nutrient medium [50] in 25 mL polypropylene tubes. A positive result was recorded as the presence of a ring of staining at the interface between air and medium. Swarming motility was tested as described by Connelly *et al.* [51] using Luria Bertani (LB) agar (Merck) containing 0.7% (*w*/*v*) agar. Each assay was performed in triplicate.

### 4.8. Evaluation of Adherence to Intestinal Epithelial Cells

#### 4.8.1. Cell Culture

The HT-29 colonic epithelial cell line was obtained from the American Type Culture Collection (ATCC, LGC Standards, Middlesex, UK). Cells were routinely grown in McCoy’s 5A modified medium supplemented with 10% fetal bovine serum (CC medium). All cells were routinely maintained in 75 cm^2^ tissue culture flasks and incubated at 37 °C in a humidified atmosphere with 5% (*v*/*v*) CO_2_. Cells were passaged when they reached ~90% confluence. 

#### 4.8.2. Assessment of Bacterial Growth in Cell Culture Medium

Overnight cultures of each *Bacillus* isolate (1 mL) were centrifuged at 18,620× *g* for 10 min and the pellet was washed and re-suspended in 1 mL CC medium. Growth curves were then performed by inoculating this bacterial suspension (1%) into 5 mL CC medium, incubating at 37 °C with agitation and measuring the OD_600_ every 2 h for 10 h and then at 24 h.

#### 4.8.3. Adherence Assays

Suspensions of each of the marine *Bacillus*, the *Bacillus* isolated from Bioplus 2B^®^ and the *Lb. rhamnosus* GG probiotic were prepared in CC medium as outlined above and the OD_600_ was adjusted in order to obtain 10^8^ CFU/mL. HT-29 cells were seeded into 12 well-plates at a density of 1 × 10^5^ cells/well. Cells were allowed to grow for 48 h and the medium was changed at least 24 h prior to addition of bacteria. Each well was then inoculated with 500 µL bacterial suspension, yielding 5 × 10^7^ CFU/well. A 500 µL aliquot of each bacterial suspension was also added to a 25 mL polypropylene tube containing 500 µL of CC medium to calculate the total CFU/mL after the incubation period. All plates and tubes were incubated in a humidified atmosphere with 5% (*v*/*v*) CO_2_ at 37 °C for 4 h. After the incubation period, the HT-29 cells were washed five times with phosphate buffered saline (PBS) to remove any unattached bacteria. The cell-associated bacteria were determined by lysing the HT-29 cells with Triton X-100 (0.1%) in PBS at 37 °C for 30 min. The number of bacterial cells per mL of CC medium was then determined by diluting the well and tube contents 10-fold in MRD and spread-plating appropriate dilutions on BHI agar incubated at 37 °C for 24 h. The experiment was conducted in triplicate. The number of bacterial cells adhering to 100 HT-29 cells was calculated. Percent adherence was also calculated as follows:

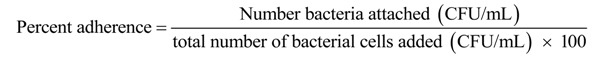
(2)


### 4.9. Analysis of Virulence Factors and Antibiotic Resistance

The *Bacillus* isolates were PCR-screened for the presence of the genes *hbl* (C, D, A, and B), *nhe* (A, B, and C), *bceT* and *cytK*, which encode the following *B. cereus* enterotoxins, respectively; hemolytic enterotoxin BL, non-hemolytic enterotoxin Nhe, bc-D-ENT enterotoxin and cytotoxin K. This was performed as described by Guinebretiere *et al.* [52], except that each 15 µL PCR reaction mixture contained 7.5 µL GoTaq^®^ Green Master Mix (Promega, Southampton, UK), 25 ρmoles/µL of both primers and 1 µL of bacterial cells adjusted to OD_600_ = 1 (NA-1000 NanoDrop^®^, Thermo Scientific, Wilmington, DE, USA). *B. cereus* DSM 31 and *B. cereus* DSM 4384 were used as positive controls, and *E. coli* DSMZ 10720 was used as a negative control. Hemolytic activity was investigated by streaking isolates in triplicate onto Columbia agar (Sigma Aldrich) with 5% sheep blood (TCS Biosciences Ltd., Buckingham, UK). *B. subtilis* PY79 and *B. cereus* DSM 31 were used as negative and positive controls, respectively. Plates were incubated at 30 °C for 3 days and examined for β-hemolysis (clear zones), α-hemolysis (greening) or γ-hemolysis (no change).

The antibiotic susceptibility of the *Bacillus* isolates was tested by determining the MIC for a panel of nine antimicrobials using a broth dilution method with commercial microtitre plates (Sensititre, TREK Diagnostic Systems Ltd., East Grinstead, UK). The microtitre wells were inoculated according to the Clinical and Laboratory Standards Institute (CLSI) guidelines [53] and incubated aerobically with agitation at room temperature for 20 h. The MIC was defined as the lowest concentration of antimicrobial that produced no visible growth. Isolates were tested with the following antimicrobials and testing ranges, as recommended by EFSA [24]; clindamycin (0.12–4 μg/mL), chloramphenicol (4–64 μg/mL), erythromycin (0.25–8 μg/mL), gentamicin (1–16 μg/mL), kanamycin (4–64 μg/mL), quinupristin/dalfopristin (0.5–4 μg/mL), streptomycin (4–32 μg/mL), tetracycline (0.5–16 μg/mL) and vancomycin (1–16 μg/mL). An isolate was considered resistant to a given antimicrobial if the MIC was higher than the breakpoint recommended by EFSA [24].

### 4.10. Evaluation of Cytotoxicity against Intestinal Epithelial Cells

Cytotoxicity of the bacterial strains against HT-29 cells was first evaluated using the trypan blue exclusion assay as follows; the HT-29 cells were grown as outlined above and suspensions of the marine and Bioplus 2B^®^ *Bacillus* and *Lb. rhamnosus* GG containing 10^8^ CFU/mL were prepared in CC medium as detailed above. One hundred microlitres of each bacterial suspension was then added to 10 mL CC medium, yielding a suspension containing 10^8^–10^9^ CFU/mL and this was added to the HT-29 cells which had been grown in 25 cm^2^ tissue culture flasks to ~90% confluence. HT-29 cells without bacteria served as a control. The flasks were then incubated in a humidified atmosphere with 5% CO_2_ at 37 °C for 5 h. After this incubation period the cells were washed five times with PBS and trypsinized to form a cell suspension. Trypan blue was added to an aliquot of this cell suspension and the number of dead (stained) and viable (unstained) cells were counted using a Countess^®^ automated cell counter (Invitrogen, Bio-Sciences, Dun Laoghaire, Co Dublin, Ireland). The experiment was conducted in triplicate. Percent cell viability was calculated as follows:

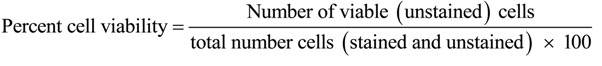
(3)


The xCELLigence real-time cell analysis system (Roche Applied Science, West Sussex, UK) was then used to obtain more detailed data on any potential toxic effects against HT-29 cells. The optimal concentration of HT-29 cells for use in assays was first determined by serially diluting HT-29 cells to final concentrations of 40,000, 20,000, 10,000, 5000, 2500, 1250 and 625 cells/mL in CC medium and seeding in triplicate into xCELLigence E-plates. The E-plates were then inserted into the xCELLigence system and incubated in a humidified atmosphere with 5% CO_2_ at 37 °C for 24 h. Impedance (CI) measurements were taken every 30 min. CI values were plotted against time and the doubling time and slope of each curve was determined for each cell concentration.

HT-29 cells were then seeded into E-plates at a density of 2 × 10^4^ cells/well (the optimal concentration determined above). The E-plates were inserted into the xCELLigence system and incubated for 20–24 h with 5% CO_2_ at 37 °C. Cell index readings were taken every 30 min. Cell pellets from overnight cultures of each of the marine *Bacillus*, the Bioplus 2B^®^ *Bacillus*, *Lb. rhamnosus* GG and two pathogenic strains (MSSA and *E. coli* O157:H7 NCTC 12900) were washed twice with CC medium and re-suspended at a concentration of 10^8^ CFU/mL in CC medium. Wells containing HT-29 cells were then inoculated (1%) with these bacterial cell suspensions in triplicate and the E-plates were incubated for a further 28 h, with CI readings taken every 10 min. The experiment was conducted in triplicate. For each bacterial strain, the CI relative to the control without bacteria (which was set at 100%) was calculated after 12 and 24 h as follows:

CI relative = (CI treatment/CI control) × 100
(4)

### 4.11. Statistical Analysis

For the anaerobic, antimicrobial activity in liquid culture and adhesion assays, the individual bacterium was considered the experimental unit. Bacterial counts were log-transformed to the base 10 prior to analysis in an attempt to ensure normal distribution. Only data which were normally distributed and with equal variances were analyzed as a one-factor analysis of variance (ANOVA) using the mixed models of SAS [54]. Cytotoxicity data were analyzed as repeated measures using the mixed procedure of SAS with time (12 and 24 h) as the repeated variable and the individual well considered the experimental unit. The appropriate covariance structure, as indicated by the model fit statistics, was fitted to the data. The denominator degrees of freedom were computed using the Satterthwaite approximation. The fixed effect was strain and replication was included as a random effect. Simple main effects were obtained using the “slide” option in SAS. Least squares means were computed and *p* values were adjusted for multiple comparisons using the Tukey-Kramer adjustment. The level of significance for all tests was *p* < 0.05.

## 5. Conclusions

Taken together, the data from the present study indicate that at least one of the marine *Bacillus* (*B. pumilus* WIT 588) has potential for use as a livestock probiotic. The strain satisfied a number of the necessary *in vitro* criteria, including antimicrobial production (mainly anti-*E. coli* activity), tolerance to simulated intestinal transit and a lack of toxic effects. Some of the other marine isolates also look promising but hemolytic activity would most likely limit their use as probiotics. The work described in the present study is only the first step in characterizing these marine *Bacillus* for use as probiotics and, while it provides important information which can be used for strain selection purposes, safety and efficacy can only be definitively determined in animal-feeding trials. A preliminary feeding trial has recently been performed in pigs with the *B. pumilus* WIT 588 strain and the data published in Prieto *et al.* [40].

## Supplementary Files

Supplementary File 1 Supplementary Information (PDF, 573 KB)
