# Supplementary material for: In Vitro Assessment of Marine Bacillus for Use as Livestock Probiotics
Source: Mar Drugs. 2014 Apr 30;12(5):2422–45. doi: 10.3390/md12052422 (PMC4052298; doi:10.3390/md12052422)
Supplement: Supplementary File 1 — Supplementary Information (PDF, 573 KB) [file marinedrugs-12-02422-s001.pdf]

Supplementary Information

Table S1. Antimicrobial activity of culture supernatants of marine *Bacillus* and *Bacillus* isolated from the Bioplus 2B® animal probiotic product against *Lactobacillus* and *Bifidobacterium*, as determined by the well diffusion assay [a].

Indicator strain	Test strain							
	***B. pumilus* WIT 582**	***B. pumilus* WIT 584**	***B. licheniformis* WIT 586**	***B. pumilus* WIT 588**	***B. pumilus* WIT 590**	***B. pumilus* WIT 592**	***B. licheniformis* DSM 5749**	***B. subtilis* DSM 5750**
Lactobacillus acidophilus LMG 9433	-	-	-	-	-	-	-	+++
Lb. rhamnosus LMG 6400	-	-	-	-	-	-	-	-
Weissella viridescens LMG 3507	-	-	-	-	-	-	+++	++++
Lb. jensenii LMG 6414	-	-	-	-	-	-	+	++++
Lb. gasseri LMG 9203	+	+	-	+	++	++	+	++
Lb. plantarum LMG 6907	-	-	-	-	-	-	-	-
Lb. casei LMG 6904	-	-	-	-	-	-	-	+++
Lb. delbrueckii subsp. lactis LMG 7942	++	+	++	++	++	++	+++	++++
Lb. buchneri LMG 6892	-	-	-	-	-	-	-	++++
Lb. amylovorus LMG 9496	++	++	+++	++	+++	++	+++	++++
Lb. paracasei ssp. paracasei LMG 7955	-	-	-	-	-	-	-	-
Lb. salivarius LMG 9477	-	-	-	-	-	-	-	-
Lb. agilis LMG 9186	-	-	-	-	-	-	+	++++
Lb. murinus LMG 14189	-	-	-	-	-	-	-	++
Lb. johnsonii DSM 10533	++	++	+++	++	+++	++	+++	++++
Lb. fermentum LMG 6902	-	-	-	-	-	-	++	+++
Lb. gallinarum LMG 9435	-	-	-	-	-	-	-	+++
Lb. rhamnosus GG	-	-	-	-	-	-	-	-
L. bulgaricus LMG 6901	+++	+++	++++	+++	++	+++	++++	++++
Bifidobacterium infantis NCIMB 702256	-	-	-	-	-	-	-	-
B. psychroaerophilum LMG 21775	-	-	-	-	-	-	-	++++

[a] Mean radii of zones of inhibition from triplicate well diffusion assays; + = 0.1–1 mm; ++ = 1.1–2 mm; +++ = 2.1–3 mm; ++++ >3 mm; - = no antimicrobial activity.

Table S2. Bacterial strains used in this study as indicators for detection of antimicrobial activity and as controls for hemolysis, enterotoxin gene, adhesion and cytotoxicity assays.

Bacterial strain	Use in this study	Growth medium	Incubation temperature (°C)	Growth conditions
Bacillus cereus DSM 31	Positive control for hemolysis and enterotoxin gene assays	BHI [a]	37	Aerobic, 200 rpm [d]
Bacillus subtilis PY79	Negative control for hemolysis assays	BHI	37	Aerobic, 200 rpm
Bacillus cereus DSM 4348	Positive control for enterotoxin gene assays	BHI	37	Aerobic, 200 rpm
Escherichia coli DSM 10720	Indicator for antimicrobial assays	BHI	37	Aerobic
Escherichia coli 0147 K88 F18	Indicator for antimicrobial assays	BHI	37	Aerobic
Escherichia coli 0141 F18ab	Indicator for antimicrobial assays	BHI	37	Aerobic
Escherichia coli F1L3	Indicator for antimicrobial assays	BHI	37	Aerobic
Escherichia coli F2S2	Indicator for antimicrobial assays	BHI	37	Aerobic
Escherichia coli F3P3	Indicator for antimicrobial assays	BHI	37	Aerobic
Escherichia coli F15OF3	Indicator for antimicrobial assays	BHI	37	Aerobic
Escherichia coli O157:H7 NCTC 12900	Positive control for cytotoxicity assay	BHI	37	Aerobic
Salmonella Typhimurium DPC 6046 (DT 104)	Indicator for antimicrobial assays	BHI	37	Aerobic
Salmonella Typhimurium DPC 6465 (PT 12)	Indicator for antimicrobial assays	BHI	37	Aerobic
Salmonella Typhimurium WIT 386 (DT 104)	Indicator for antimicrobial assays	BHI	37	Aerobic
Salmonella Typhimurium WIT 397 (DT 17)	Indicator for antimicrobial assays	BHI	37	Aerobic
Salmonella Derby WIT 411	Indicator for antimicrobial assays	BHI	37	Aerobic
Vibrio cholerae N16961	Indicator for antimicrobial assays	LB + NaCl [b]	30	Aerobic
Vibrio fischeri MJ11	Indicator for antimicrobial assays	LB + NaCl	30	Aerobic
Methicillin sensitive *Staphylococcus aureus* (MSSA)	Positive control for cytotoxicity assay	BHI	37	Aerobic
Lactobacillus rhamnosus GG (LMG 6400)	Indicator, control for adherence and cytotoxicity assays	MRS + cysteine [c]	37	Anaerobic
Lactobacillus acidophilus LMG 9433	Indicator for antimicrobial assays	MRS + cysteine	37	Anaerobic
Lactobacillus rhamnosus LMG 6400	Indicator for antimicrobial assays	MRS + cysteine	37	Anaerobic
Lactobacillus jensenii LMG 6414	Indicator for antimicrobial assays	MRS + cysteine	37	Anaerobic
Lactobacillus gasseri LMG 9203	Indicator for antimicrobial assays	MRS + cysteine	37	Anaerobic
Lactobacillus plantarum LMG 6907	Indicator for antimicrobial assays	MRS + cysteine	37	Anaerobic
Lactobacillus casei LMG 6904	Indicator for antimicrobial assays	MRS + cysteine	37	Anaerobic

Table S2. *Cont.*

Bacterial strain	Use in this study	Growth medium	Incubation temperature (℃)	Growth conditions
Lactobacillus delbrueckii subsp. lactis LMG 7942	Indicator for antimicrobial assays	MRS + cysteine	37	Anaerobic
Lactobacillus buchneri LMG 6892	Indicator for antimicrobial assays	MRS + cysteine	37	Anaerobic
Lactobacillus amylovorus LMG 9496	Indicator for antimicrobial assays	MRS + cysteine	37	Anaerobic
Lactobacillus paracasei ssp. paracasei LMG 7955	Indicator for antimicrobial assays	MRS + cysteine	37	Anaerobic
Lactobacillus salivarius LMG 9477	Indicator for antimicrobial assays	MRS + cysteine	37	Anaerobic
Lactobacillus agilis LMG 9186	Indicator for antimicrobial assays	MRS + cysteine	37	Anaerobic
Lactobacillus murinus LMG 14189	Indicator for antimicrobial assays	MRS + cysteine	37	Anaerobic
Lactobacillus johnsonii DSM 10533	Indicator for antimicrobial assays	MRS + cysteine	37	Anaerobic
Lactobacillus fermentum LMG 6902	Indicator for antimicrobial assays	MRS + cysteine	37	Anaerobic
Lactobacillus gallinarum LMG 9435	Indicator for antimicrobial assays	MRS + cysteine	37	Anaerobic
Lactobacillus bulgaricus LMG 6901	Indicator for antimicrobial assays	MRS + cysteine	37	Anaerobic
Bifidobacterium infantis NCIMB 702256	Indicator for antimicrobial assays	MRS + cysteine	37	Anaerobic
Bifidobacterium psychroaerophilum LMG 21775	Indicator for antimicrobial assays	MRS + cysteine	37	Anaerobic
Weissella viridescens LMG 3507	Indicator for antimicrobial assays	MRS + cysteine	37	Anaerobic

[a] Brain heart infusion (BHI; Oxoid, Basingstoke, Hampshire, UK); [b] Luria Bertani Miller (LB; Merck, Darmstadt, Germany) supplemented with 3% (*w*/*v*) NaCl; [c] de Man, Rogosa and Sharpe (MRS) ((Becton, Dickinson and Company (BD), Franklin Lakes, NJ, USA) supplemented with 0.05% (*w*/*v*) L-cysteine; [d] Broths were incubated with shaking at 200 rpm.

Figure S1. Influence of cell density on impedance (cell index) measurements taken using the xCELLigence real-time cell analysis system. HT-29 cells at densities of (**A**) 40,000; (**B**) 20,000; (**C**) 10,000; (**D**) 5000; (**E**) 2500; (**F**) 1250 and (**G**) 625 cells/mL in E-Plates were observed for 48h. Values are the mean of data from triplicate assays conducted on the same day, with SE indicated by error bars.

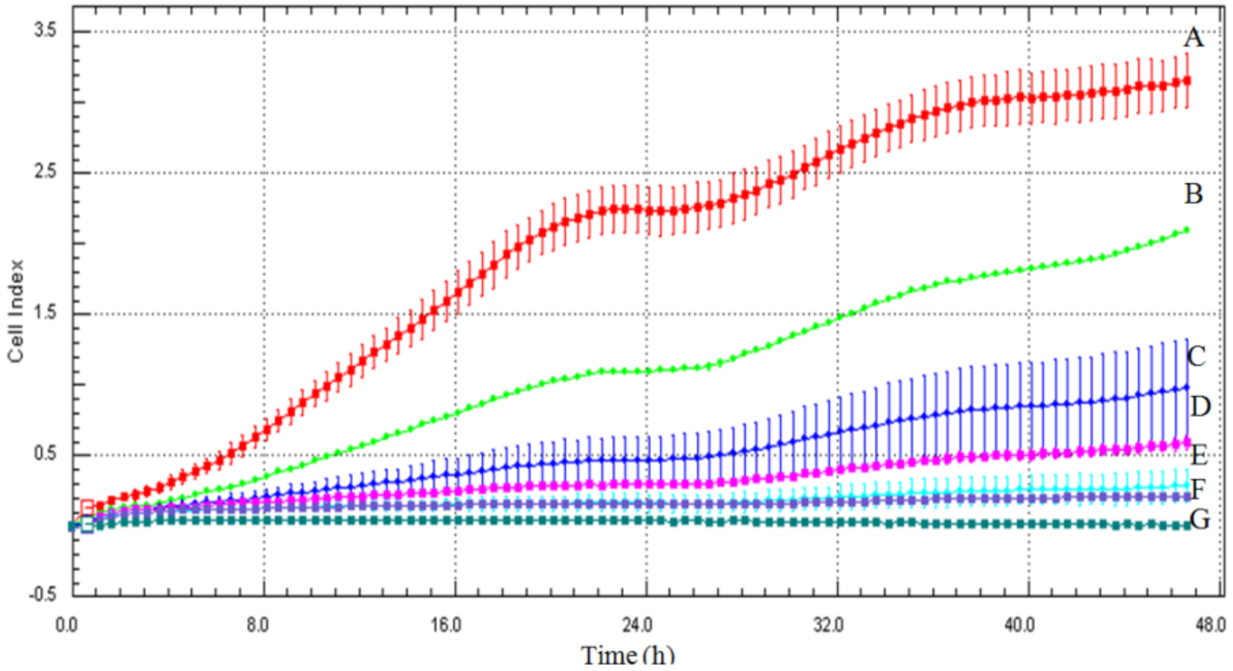

Figure S2. Dynamic monitoring of the influence of bacterial addition on impedance (cell index) measurements taken using the xCELLigence real-time cell analysis system. HT-29 cells at a density of 20,000 cells/mL were seeded into E-plates alone (control) or together with the bacterial strains indicated.

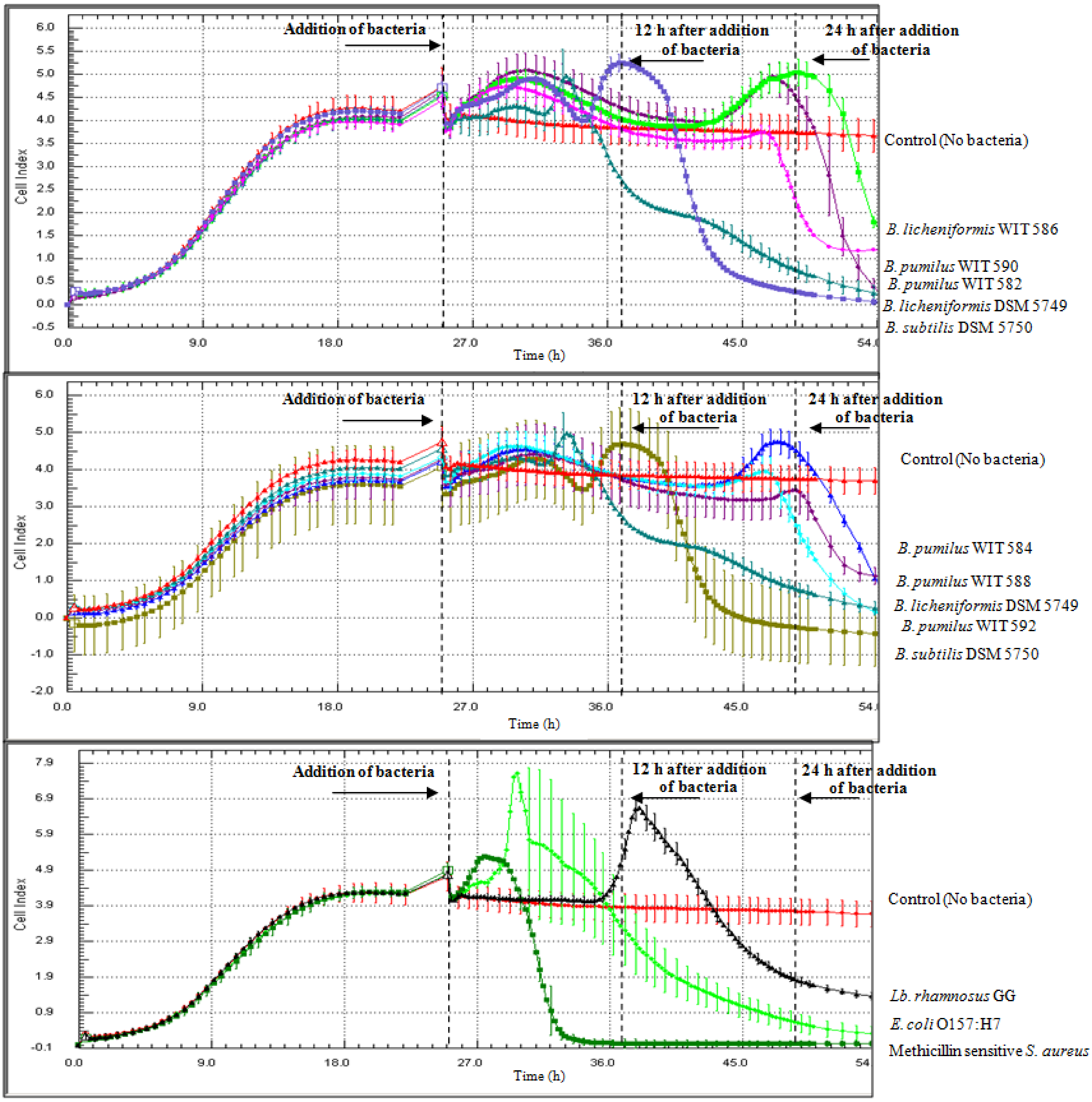

© 2014 by the authors; licensee MDPI, Basel, Switzerland. This article is an open access article distributed under the terms and conditions of the Creative Commons Attribution license (http://creativecommons.org/licenses/by/3.0/).
